# Supplementary material for: CAS12e (CASX2) CLEAVAGE OF CCR5: IMPACT OF GUIDE RNA LENGTH AND PAM SEQUENCE ON CLEAVAGE ACTIVITY
Source: bioRxiv. 2023 Jan 2:2023.01.02.522476. Preprint. [Version 1] doi: 10.1101/2023.01.02.522476 (PMC9881857; doi:10.1101/2023.01.02.522476)
Supplement: Supplement 1 — Supplemental Figure 1. The 1,000 bp region of the CCR5 gene containing all sgRNA target sequences. Complete sequence of the CCR5 gene region (Chr3:46372947–46373940) within exon 2 on chromosome 3 displaying the location of each sgRNA relative to the location that would be deleted in the Δ−32 mutation (blue rectangle). sgRNAs are shown in red and the protospacer adjacent motifs (PAM) in gray. Supplemental Figure 2. Cleavage activity by Cas12e (CasX2) is gRNA spacer length and DNA target location dependent. Examples of agarose gel separation of CasX2 cleavage products by nine different sgRNAs are shown (sg2, sg3, sg4, sg6, sg9, sg1, sg5, sg8, and sg10). The number and size of the cleavage products are associated with their relative location within the 2,812 nt target region. Shown is one representative experiment for each gRNA at all spacer lengths. All experiments were run in triplicate. Supplemental Figure 3. Terminal PAM base impacts Cas12e cleavage activity for multiple sgRNAs. CCR5 DNA targets were generated to contain either an A, G, C or T in the terminal PAM location for each of the sgRNAs. Targets underwent in vitro cleavage to assess the impact of altering the terminal PAM base. A consistent cleavage pattern among several gRNAs showed the highest cleavage activity with an A or G as the terminal PAM base. To aid in visualizing cleavage products, different sized CCR5 targets were utilized dependent on the guide location. For sgRNA 3 (panel A), a SpeI/XhoI 1,720 bp target was used, for sgRNA 4 (panel B) and sgRNA 7 (panel C), a NheI/XhoI 1,074 bp target was used, and for sgRNA 10 (panel D), a NheI/SmaI 2,166 bp target was used. Arrows indicate the location of the uncleaved target. [file media-1.pdf]

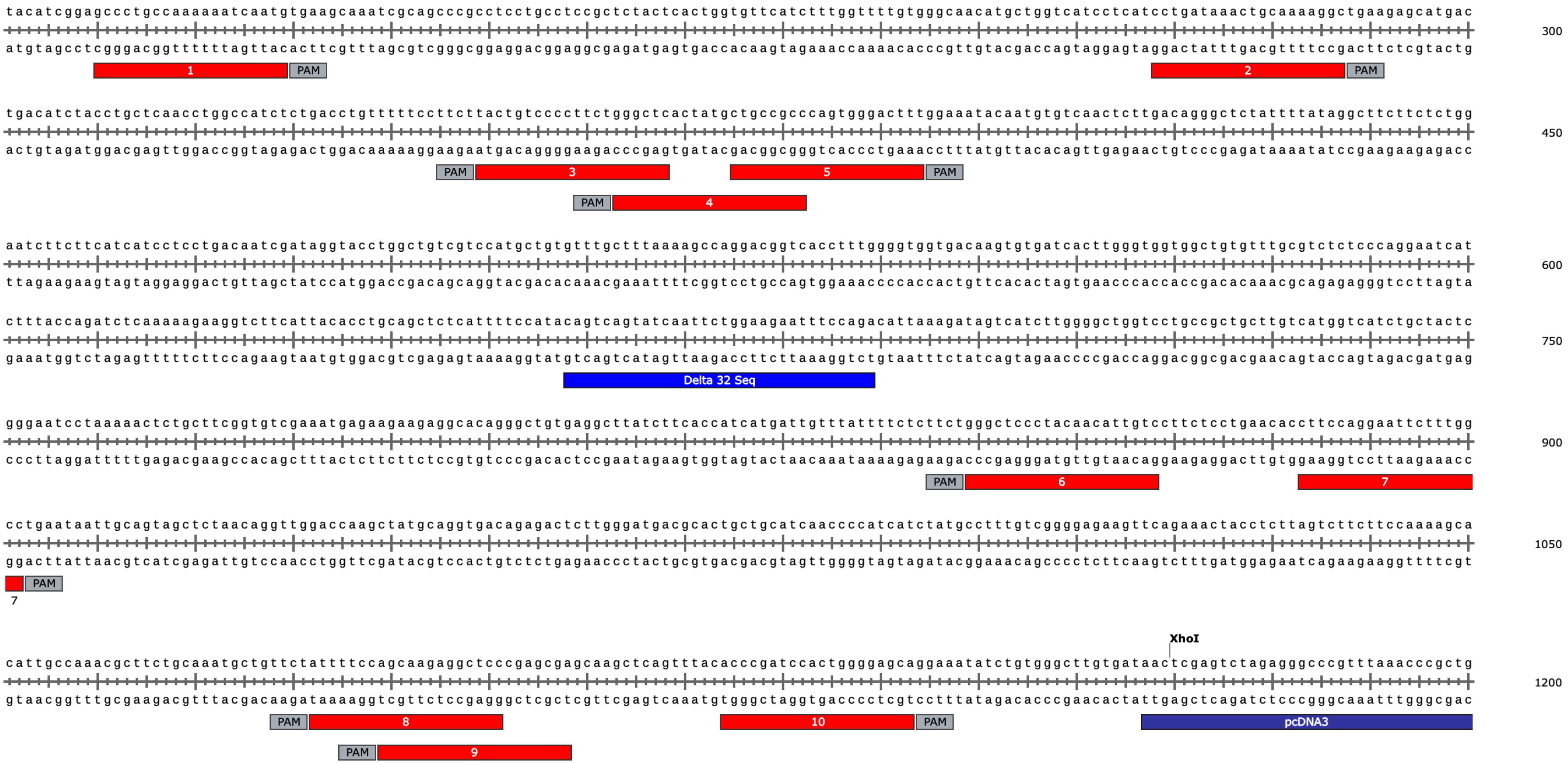

Supplemental Figure 1.

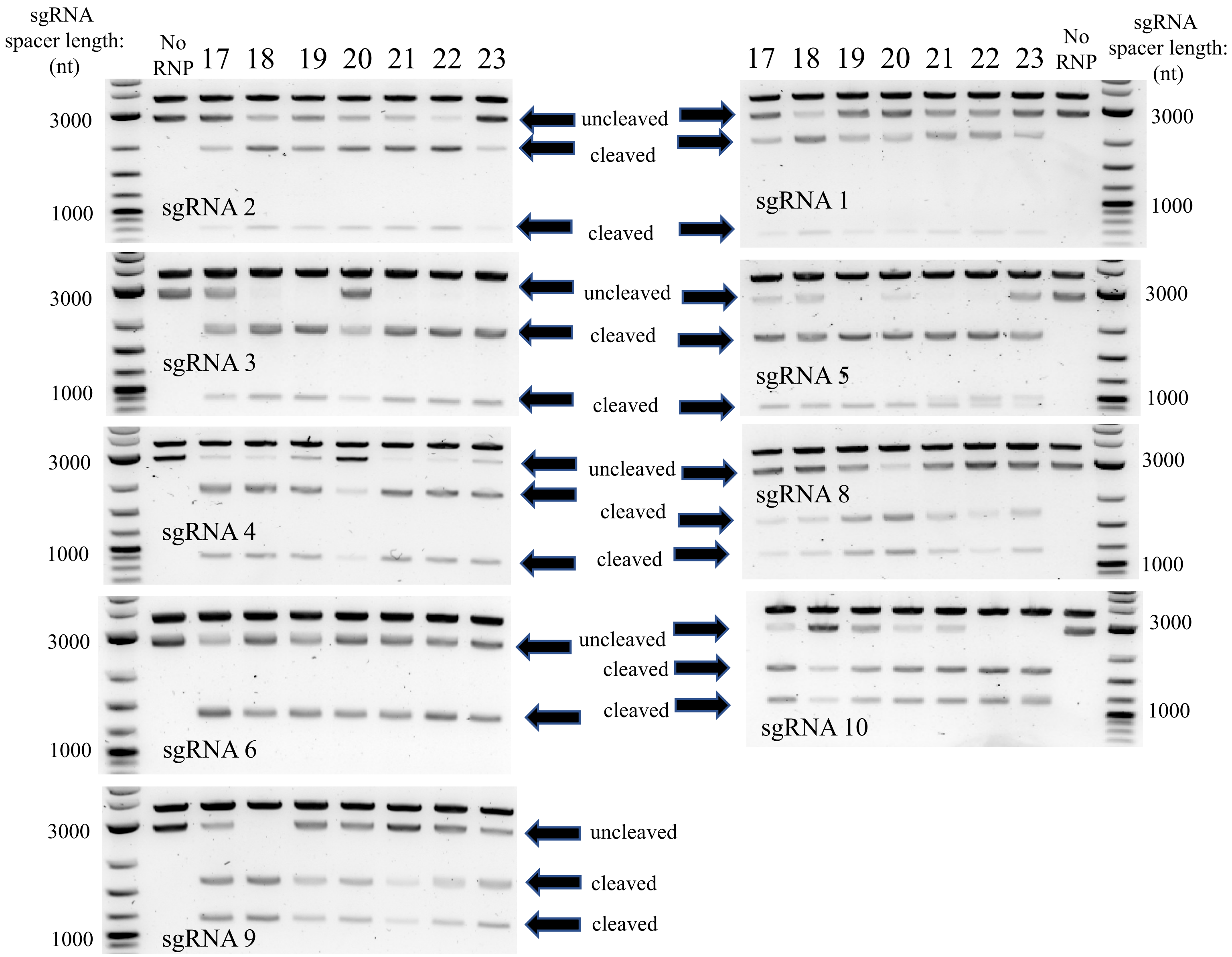

Supplemental Figure 2.

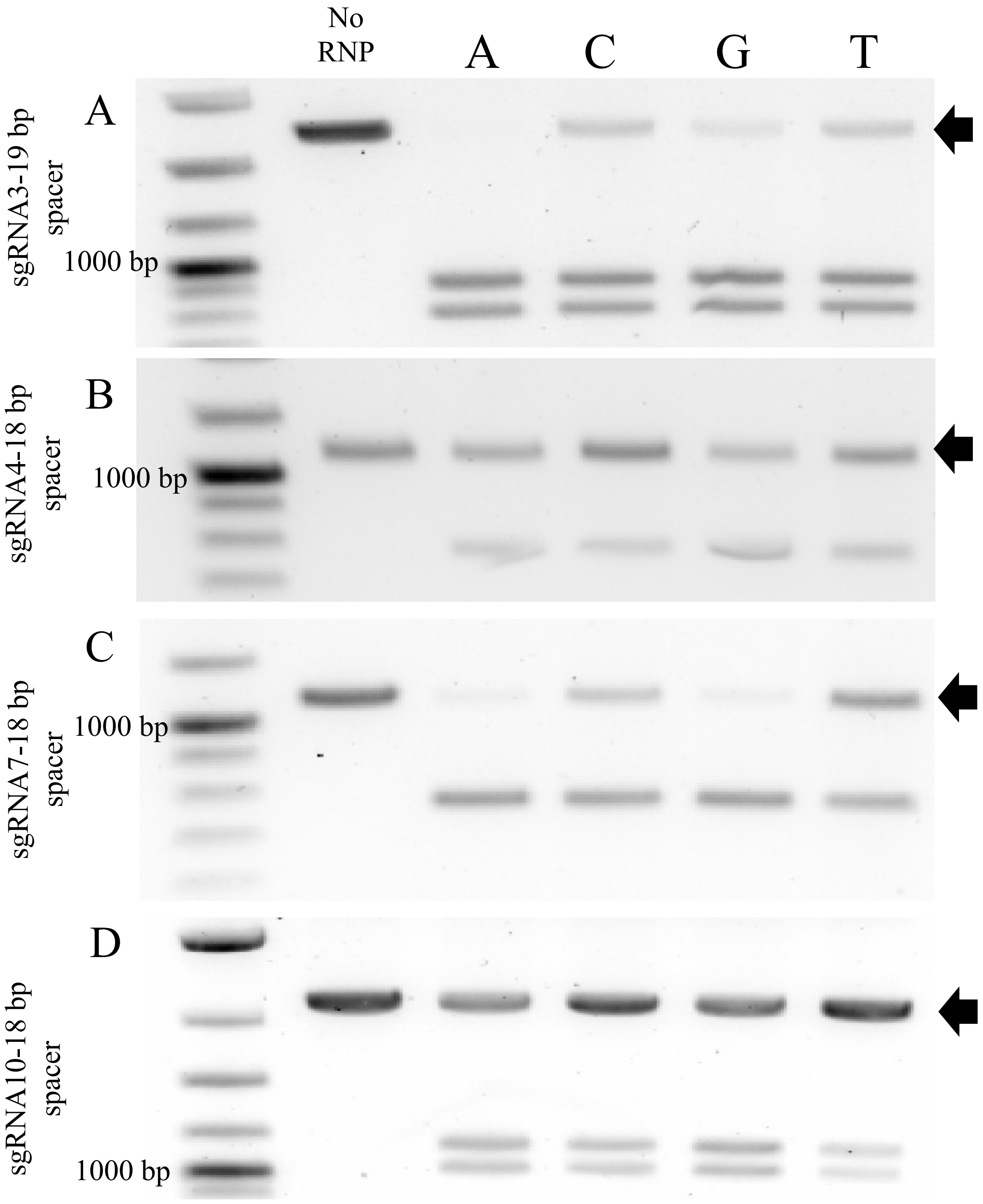

Supplemental Figure 3.

Supplemental Table 1. **DNA OLIGOS for in vitro transcription of guide RNAs**

Scaffold DNA Oligo

5’- gaaatTAATACGACTCACTATAGTACTGGCGCTTTTATCTCATTACTTTGAGAGCCATCACCAGCGACTATGTCGTATGGGTAAAGCGCTTATTTATCGGAGAGAAATCC - 3'

TRACR RNA 5'-GTACTGGCGCTTTTATCTCATTACTTTGAGAGCCATCACCAGCGACTATGTCGTATGGGTAAAGCGCTTATTTATCGGAGA(81)

BLACK - T7 PRIMER

RED - TRACR

GREEN - LOOP/LINKER

Reverse DNA Oligo

3’- ATAAATAGCCTCTCTTTAGGCTATTTATTCTTCGTAGTTTCNNNNNNNNNNNNNNNNNNNN-5’

PURPLE - (NNN's) - GUIDE REVERSE OLIGOS (17-23 BP)

BLUE - REPEAT

GREEN - LOOP/LINKER COMPLEMENT

Supplemental Table 2. CCR5 sgRNA characteristics

Index	% Target Cleavage
(++++)	90-100
(+++)	89-70
(++)	69-50
(+)	<50

sgRNA Number	Spacer Length	Guide Sequence	Terminal RNA Base	% G/C	PAM	Cutting Index	delta G (kcal/mol)
1	17	5'-CAUUGAUUUUUUGGCAG	G	35	TTCA	(+)	-31.81
	18	5'-CAUUGAUUUUUUGGCAGG	G	39		(++)	-34.88
	19	5'-CAUUGAUUUUUUGGCAGGG	G	42		(+)	-37.95
	20	5'-CAUUGAUUUUUUGGCAGGGC	C	45		(+)	-41.09
	21	5'-CAUUGAUUUUUUGGCAGGGCU	U	43		(++)	-42.69
	22	5'-CAUUGAUUUUUUGGCAGGGCUC	C	45		(++)	-44.26
	23	5'-CAUUGAUUUUUUGGCAGGGCUCC	C	48		(+)	-47.33
2	17	5'-GCCUUUUGCAGUUUAUC	C	41	TTCA	(+)	-31.53
	18	5'-GCCUUUUGCAGUUUAUCA	A	39		(++)	-33.48
	19	5'-GCCUUUUGCAGUUUAUCAG	G	43		(+)	-35.08
	20	5'-GCCUUUUGCAGUUUAUCAGG	G	45		(++)	-38.15
	21	5'-GCCUUUUGCAGUUUAUCAGGA	A	43		(++)	-39.72
	22	5'-GCCUUUUGCAGUUUAUCAGGAU	U	41		(+++)	-41.2
	23	5'-GCCUUUUGCAGUUUAUCAGGAUG	G	43		(+)	-43.15
3	17	5'-UACUGUCCCCUUCUGGG	G	59	TTCT	(+++)	-32.79
	18	5'-UACUGUCCCCUUCUGGGC	C	61		(++)	-35.93
	19	5'-UACUGUCCCCUUCUGGGCU	U	58		(+++)	-37.53
	20	5'-UACUGUCCCCUUCUGGGCUC	C	60		(+++)	-39.1
	21	5'-UACUGUCCCCUUCUGGGCUCA	A	57		(++++)	-41.06
	22	5'-UACUGUCCCCUUCUGGGCUCAC	C	59		(++++)	-42.4
	23	5'-UACUGUCCCCUUCUGGGCUCACU	U	57		(++)	-44
4	17	5'-AAAGUCCCACUGGGCGG	G	65	TTCC	(+++)	-37.35
	18	5'-AAAGUCCCACUGGGCGGC	C	67		(++++)	-40.49
	19	5'-AAAGUCCCACUGGGCGGCA	A	63		(+++)	-42.44
	20	5'-AAAGUCCCACUGGGCGGCAG	G	65		(+)	-44.04
	21	5'-AAAGUCCCACUGGGCGGCAGC	C	67		(+++)	-47.18
	22	5'-AAAGUCCCACUGGGCGGCAGCA	A	64		(+++)	-49.13
	23	5'-AAAGUCCCACUGGGCGGCAGCAU	U	61		(+++)	-50.61
5	17	5'-GGGCUCACUAUGCUGCC	C	65	TTCT	(+++)	-34.64
	18	5'-GGGCUCACUAUGCUGCCG	G	67		(++)	-38.25
	19	5'-GGGCUCACUAUGCUGCCGC	C	68		(+++)	-41.39
	20	5'-GGGCUCACUAUGCUGCCGCC	C	70		(+++)	-44.46
	21	5'-GGGCUCACUAUGCUGCCGCCC	C	71		(++++)	-47.52
	22	5'-GGGCUCACUAUGCUGCCGCCCA	A	68		(++++)	-49.48
	23	5'-GGGCUCACUAUGCUGCCGCCCAG	G	70		(++)	-51.08

6	17	5'-GGGCUCCCUACAACAUU	U	53	TTCT	(+)	-33.1
	18	5'-GGGCUCCCUACAACAUUG	G	56		(+)	-35.06
	19	5'-GGGCUCCCUACAACAUUGU	U	53		(+++)	-36.4
	20	5'-GGGCUCCCUACAACAUUGUC	C	55		(++)	-37.98
	21	5'-GGGCUCCCUACAACAUUGUCC	C	57		(+)	-41.04
	22	5'-GGGCUCCCUACAACAUUGUCCU	U	55		(+)	-42.64
	23	5'-GGGCUCCCUACAACAUUGUCCUU	U	52		(+)	-44.59
7	17	5'-GCCCAAAGAAUUCCUGG	G	53	TTCA	(++++)	-34.92
	18	5'-GGCCAAAGAAUUCCUGGA	A	50		(++++)	-36.5
	19	5'-GGCCAAAGAAUUCCUGGAA	A	47		(++++)	-38.44
	20	5'-GGCCAAAGAAUUCCUGGAAG	G	50		(++++)	-40.04
	21	5'-GGCCAAAGAAUUCCUGGAAGG	G	52		(++)	-43.11
	22	5'-GGCCAAAGAAUUCCUGGAAGGU	U	50		(++)	-44.45
	23	5'-GGCCAAAGAAUUCCUGGAAGGUG	G	52		(++)	-46.41
8	17	5'-AUUUUCCAGCAAGAGGC	C	47	TTCT	(+)	-33.52
	18	5'-AUUUUCCAGCAAGAGGCU	U	44		(+)	-35.12
	19	5'-AUUUUCCAGCAAGAGGCUC	C	47		(++)	-36.7
	20	5'-AUUUUCCAGCAAGAGGCUCC	C	50		(+++)	-39.76
	21	5'-AUUUUCCAGCAAGAGGCUCCC	C	52		(+)	-42.83
	22	5'-AUUUUCCAGCAAGAGGCUCCCG	G	55		(+)	-46.45
	23	5'-AUUUUCCAGCAAGAGGCUCCCGA	A	52		(+)	-48.02
9	17	5'-AGCAAGAGGCUCCCGAG	G	65	TTCC	(++)	-35.71
	18	5'-AGCAAGAGGCUCCCGAGC	C	67		(++++)	-38.85
	19	5'-AGCAAGAGGCUCCCGAGCG	G	68		(+)	-42.46
	20	5'-AGCAAGAGGCUCCCGAGCGA	A	65		(++)	-44.04
	21	5'-AGCAAGAGGCUCCCGAGCGAG	G	67		(+)	-45.64
	22	5'-AGCAAGAGGCUCCCGAGCGAGC	C	68		(++)	-48.78
	23	5'-AGCAAGAGGCUCCCGAGCGAGCA	A	65		(++)	-50.73
10	17	5'-UGCUCCCCAGUGGAUCG	G	65	TTCC	(+++)	-35.63
	18	5'-UGCUCCCCAGUGGAUCGG	G	67		(+)	-38.7
	19	5'-UGCUCCCCAGUGGAUCGGG	G	68		(++)	-41.77
	20	5'-UGCUCCCCAGUGGAUCGGGU	U	65		(+++)	-43.11
	21	5'-UGCUCCCCAGUGGAUCGGGUG	G	67		(+++)	-45.06
	22	5'-UGCUCCCCAGUGGAUCGGGUGU	U	64		(++++)	-46.4
	23	5'-UGCUCCCCAGUGGAUCGGGUGUA	A	61		(++++)	-47.36
